# Supplementary figures and images for: Functional characterization of SARS-CoV-2 vaccine elicited antibodies in immunologically naïve and pre-immune humans
Source: bioRxiv. 2021 May 31:2021.05.29.445137. Preprint. [Version 1] doi: 10.1101/2021.05.29.445137 (PMC8183011; doi:10.1101/2021.05.29.445137)

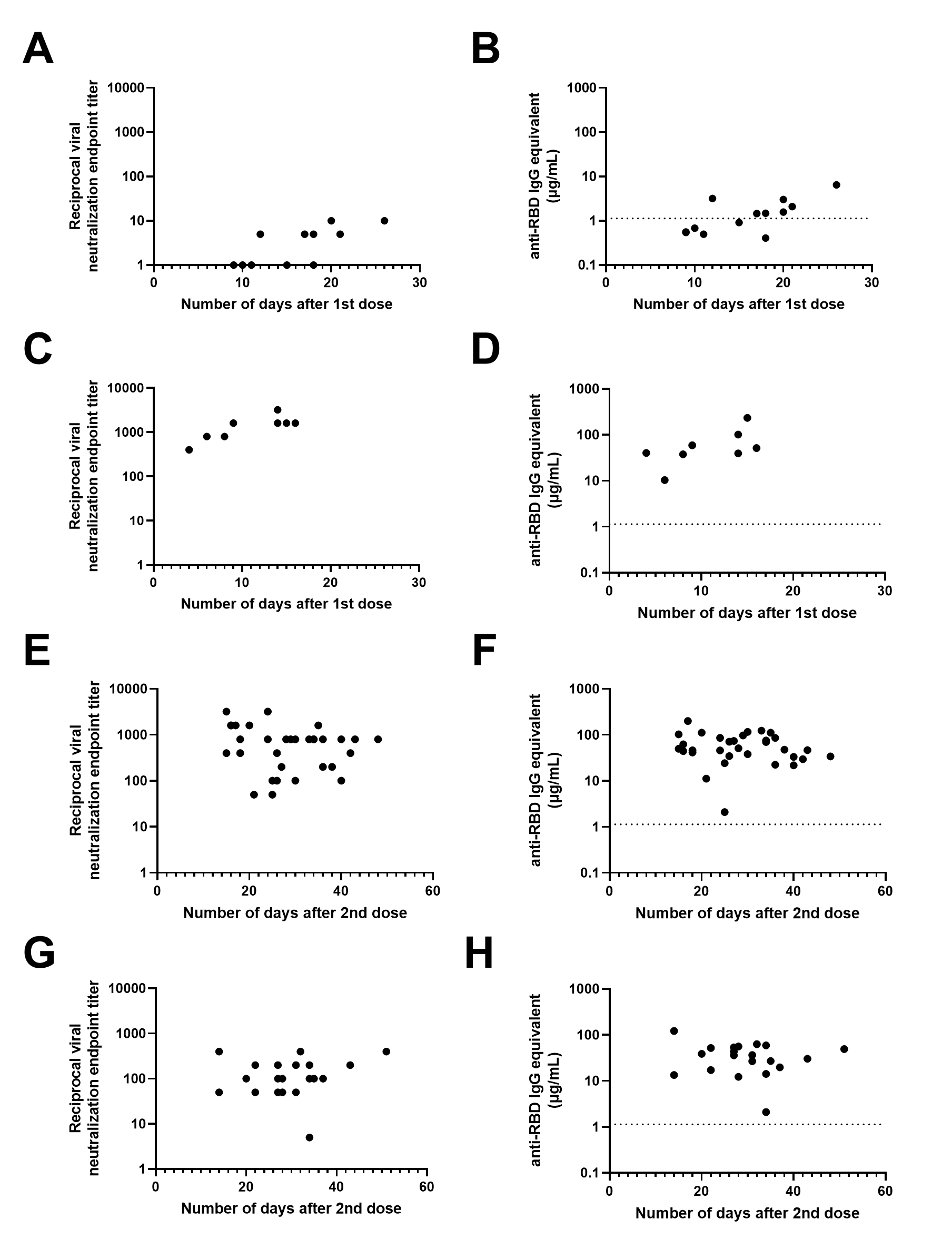

Supplement: Supplement 3 — Fig. S1: Antibody response based on number of days after the reception of the first and second vaccinations. In immunologically naïve participants, (A) neutralizing and (B) anti-RBD IgG antibody levels showed a positive correlation with the number of days after the first vaccination the serum was collected (r=0.7781, ** p=0.0017, and r=0.6861, ** p=0.0096 respectively). In infected participants, (C) neutralizing antibody levels showed no correlation, with the number of days after the first vaccination the serum was collected (r=0.5319, p=0.1749), but (D) binding antibody levels did show a slight positive correlation (r=0.7085, * p=0.0492). In immunologically naïve participants, (E) neutralizing and (F) binding antibody levels showed no correlation with the number of days after the second vaccination the serum was collected (r=−0.3313, p=0.064, and r=−0.2190, p=0.2284 respectively). Similarly, in infected participants, neither (G) neutralizing nor (H) binding antibody levels showed any correlation with the number of days after the second vaccination the serum was collected (r=0.2068, p=0.3817, and r=−0.2458, p=0.2963 respectively). In order to represent the values on a logarithmic scale, lack of neutralization was reported as 1. [file media-3.tif]
